# Umbilical Cord Structural Changes in Pregnancy-Induced Hypertension: Histological and Morphometric Insights

**DOI:** 10.7759/cureus.78951

**Published:** 2025-02-13

**Authors:** Deepti Chandrakar, Meena Goyal, Diwakar Dhurandhar, Sangeeta Khare, Kushal Chakraborty, Jagriti Agrawal

**Affiliations:** 1 Anatomy, Pt. Jawahar Lal Nehru Memorial (JNM) Medical College, Raipur, IND

**Keywords:** histomorphology, histomorphometry, intrauterine growth retardation, pregnancy induced hypertension, umbilical cord

## Abstract

Introduction

Pregnancy-induced hypertension (PIH) is a significant contributor to feto-maternal morbidity and mortality, especially in developing nations like India, where perinatal outcomes tend to be poor. This study was conducted to assess the potential histological and histomorphometric alterations in the umbilical cord of full-term mothers with normal pregnancies and those affected by PIH.

Materials and methods

In this observational, cross-sectional study, umbilical cords from 31 mothers in each of the two groups were selected according to specified inclusion and exclusion criteria: full-term mothers with normal pregnancies and those with PIH. Paraffin sections of the umbilical cords, approximately 5 µm thick, were prepared. Systematic random sampling was done to select samples for making slides and staining using hematoxylin & eosin (H&E) and periodic acid-Schiff (PAS) stains. The histology of the umbilical artery, vein, and Wharton's jelly was analyzed under a microscope. Histomorphometric parameters, specifically the wall thickness of the umbilical artery and vein, were measured. An unpaired Student's t-test was applied to determine if there was any statistically significant difference. A p-value of less than 0.05 was considered significant.

Results

On microscopic examination, the umbilical artery from the PIH Group showed a contracted lumen, thickening of the wall, increased hydropic changes, and deposition of fibrinoid material in the tunica intima and media. An umbilical vein from the PIH Group showed a dilated lumen, thinning of the wall, increased hydropic changes, increased intercellular space, contraction of cells, and disturbed architecture of smooth muscle cells (SMCs) and their nuclei, whereas Wharton’s jelly from the PIH Group exhibited increased edema and decreased cellularity in comparison to the Control Group. In histomorphometric evaluation using an unpaired Student's t-test, the thickness of the wall of the umbilical artery in the PIH Group (798.715 ± 106.34 μm) was increased compared to the Control Group (718.206 ± 75.72 μm), with a p-value of 0.0002, and the results were extremely significant, whereas the thickness of the wall of the umbilical vein was reduced in the PIH Group (478.800 ± 76.65 μm) compared to the Control Group (667.506 ± 51.31 μm). The results were extremely significant, with a p-value of less than 0.0001.

Conclusion

Increased umbilical arterial wall thickness, decreased umbilical vein wall thickness, and decreased cellularity with edema of Wharton’s jelly were observed, which could be a predictor of intrauterine growth restriction (IUGR) in term pre-eclamptic patients.

## Introduction

Pregnancy-induced hypertension (PIH) is a multisystem disorder in pregnancy, more commonly seen in first pregnancies and primarily affecting the maternal renal, cerebral, and hepatic systems [1]. PIH complicates 3%-5% of all pregnancies and is responsible for a significant proportion of maternal and fetal deaths [2]. In addition to being a leading cause of feto-maternal morbidity and mortality, PIH is associated with unfavorable perinatal outcomes [2]. Common complications include intrauterine growth restriction (IUGR), preterm delivery, low birth weight, and fetal death due to premature birth [2]. Although early prediction of PIH is challenging, certain epidemiological risk factors are known, such as nulliparity, prior preeclampsia, family history, black ethnicity, obesity, diabetes mellitus, multifetal pregnancies, maternal age (<18 and >35), and pre-existing renal disease [3].

The histology of the umbilical cord in normal pregnancies is well-established, showing a complex structure composed of Wharton's jelly, umbilical vessels, and amniotic epithelium [4,5], all of which contribute to its elasticity and strength. However, in pregnancies complicated by PIH, the umbilical cord may experience notable structural changes due to the pathophysiological effects of maternal hypertension [6-9]. Bruch et al. reported that growth-restricted fetuses, whether or not they exhibited umbilical artery Doppler abnormalities, had a smaller umbilical cord cross-sectional area at delivery compared to healthy fetuses at the same gestational age [7]. Di Naro et al. found that umbilical cord diameters and areas varied throughout gestation, with these changes primarily attributed to a reduction in Wharton's jelly rather than alterations in the umbilical vessels themselves [8]. Junek et al. observed that the umbilical arteries were thicker in preeclamptic pregnancies compared to uncomplicated pregnancies [9], particularly in the tunica intima and media. These structural changes may contribute to fetal growth restriction and poor perinatal outcomes.

Given this background, the present study aimed to investigate the histological and histomorphometric differences between the umbilical cords of normal, healthy pregnancies and those affected by PIH. By analyzing structural changes in umbilical cord tissues, the study seeks to enhance our understanding of the mechanisms by which PIH influences fetal development. The results may provide important insights into how maternal hypertension affects placental function, potentially contributing to the management of high-risk pregnancies and improving fetal outcomes.

## Materials and methods

This cross-sectional, observational study on umbilical cords obtained from mothers delivering at BRAM Hospital, Raipur, was undertaken in the Department of Anatomy, Pt. Jawahar Lal Nehru Memorial (JNM) Medical College, Raipur, India, after obtaining due approval from the Institutional Ethics Committee and informed consent from mothers.

Inclusion criteria

For the Control Group, singleton uncomplicated term pregnancy, gestational period >36 weeks (up to 40 weeks), and cases having normal blood pressure and blood sugar levels.

For the PIH Group, singleton uncomplicated term pregnancy, gestational period >36 weeks (up to 40 weeks), and mothers with blood pressure >140/90 on two or more occasions, at least six hours apart, after 20 weeks of gestation, with or without proteinuria.

Exclusion criteria

Pregnancy is complicated by other medical disorders such as diabetes mellitus, essential hypertension, chronic renal disease, and platelet disorders, as well as the umbilical cord of multiple pregnancies and the umbilical cord of pre-term pregnancies.

Sample size calculation

The sample size was calculated using the principle of estimating a population proportion with specified absolute precision. The anticipated population proportion was 1%, with a confidence level of 90% and an absolute precision of 10% (0.1).

 $n = \frac{Z^2 (1 - \alpha) P (1 - P)}{d^2}$

By using the above formula, the sample size calculated was 31 cases and 31 controls. 

Materials required for study

The umbilical cord, along with placentae, of 62 delivered patients, including 31 normal pregnancies and 31 with PIH; tray and bucket; scalpel, knife, forceps, needle, and scissors. Chemicals required are 10% formol saline, xylene, alcohol (50%, 70%, 90%, and 100%), paraffin wax, hematoxylin & eosin (H&E) stains, egg albumin, dibutyl phthalate xylene (DPX), distilled water, and constituents of periodic acid-Schiff (PAS). Other materials used were Olympus Biological Microscope - Binocular LED (MX 21i) (Olympus Corporation, Tokyo, Japan), containers, oven, microtome, microtome knife, microtome knife sharpener, slides, coverslips, slide warmer, and Coplin jar.

Variables

Demographic parameters such as age of the mother (in years), parity of the mother, gestational age (in weeks), systolic and diastolic blood pressure (mmHg), mode of delivery (lower section cesarean section or full-term vaginal delivery), APGAR (Appearance, Pulse, Grimace, Activity, Respiration) score of the newborn at the time of birth, fetal weight (in kg), gender of the newborn, and histomorphometric parameters such as wall thickness of the umbilical artery and wall thickness of the umbilical vein.

Methodology

A total of 31 mothers in each group were selected on the basis of inclusion and exclusion criteria. Written consent was obtained from the mothers after explaining the study details, and the mother’s proforma was maintained. Subsequently, 31 specimens, comprising placentae along with umbilical cords from women with normal pregnancies and 31 with PIH, were collected. Subjects were divided into two groups. Group I consisted of umbilical cords obtained from normal pregnant women (n = 31) with normal blood pressure (systolic BP <140 mmHg and diastolic BP <90 mmHg), gestational age 37-40 weeks. Group II consisted of umbilical cords obtained from PIH women. Women were diagnosed with PIH if they had systolic BP ≥140 mmHg, diastolic BP ≥90 mmHg, measured on two or more occasions at least four hours apart after the 20th week of gestation, with or without proteinuria and edema. Proteinuria was considered to be present when there was a dipstick value of at least 1+ (≥300 mg/dL) on two separate occasions at least six hours apart. A sample of the umbilical cord, along with the placenta, was collected within two hours of delivery and washed under running tap water to remove blood smears and clots. Then, a section 5 cm from the site of insertion to the placenta was taken.

After collection, umbilical cords were cut 5 cm away from the placental end and fixed in formaldehyde. Tissue sizes of 5 mm thickness were taken. Two blocks, each having a thickness of 2.5 mm, were made from each umbilical cord for studying histomorphology and histomorphometry of the umbilical cord. A routine paraffin procedure was done. Tissue samples were fixed in a 10% formalin solution. The specimens were dehydrated using a graded ethanol series, cleared with xylene, and embedded in paraffin. Sections were cut at a thickness of 5 μm, yielding approximately 500 sections. Every fifth section was selected, deparaffinized, rehydrated, and subsequently stained with H&E. Thus, systematic random samples of umbilical cord sections were identified, and a morphometric study was performed using an Olympus microscope.

Histomorphometric study

Sections of the umbilical cord were seen under a microscope at 10X, 40X, and 100X magnifications, and the following observations were made using an ocular micrometer. The wall thickness of the umbilical artery was measured as the thickness of the wall, including the thickness of the tunica intima and media, as no distinct tunica adventitia could be delineated. Similarly, the wall thickness of the umbilical vein was measured. These measurements were done at 40X and 100X magnifications.

Analysis of data

The collected data was put in the master chart. The mean and standard deviation were calculated for umbilical artery and umbilical vein wall thickness in both groups. The comparison of the means was done using Student's unpaired t-test. In all the tests, a p-value less than 0.05 was taken to be statistically significant. Simple average values were obtained by summing all the individual observations and dividing them by the number of observations.

## Results

After collection, 31 umbilical cords from each group - Control Group and PIH Group - were obtained, and the demographic characteristics of the mothers were tabulated in Table 1.

**Table 1:** Demographic findings in the Control Group and PIH Group and their p-values. PIH: Pregnancy-induced hypertension; BP: Blood pressure; FTVD: Full-term vaginal delivery; APGAR: Appearance, pulse, grimace, activity, respiration; Mch: Male children; Fch: Female children

Group	Control	PIH	p-value
Age (in years)	23.61 ± 2.95	22.42 ± 1.96	-
Parity (nulliparous/multiparous)	17/14	20/11	-
Gestational age (in weeks)	37.94 ± 1.41	37.06 ± 1.56	-
Systolic BP (mmHg)	107.94 ± 5.32	156.13 ± 28.1	0.00000406
Diastolic BP (mmHg)	69.35 ± 4.35	104 ± 4.93	0.000154
Mode of delivery, LSCS/FTVD	14/17	14/17	-
APGAR	8.77 ± 0.61	6.94 ± 1.05	0.031303
Fetal weight (in kg)	2.85 ± 0.43	2.29 ± 0.50	0.000459
Sex (Mch/Fch)	21/10	20/11	-

Histology of umbilical cord

**Figure 1:**
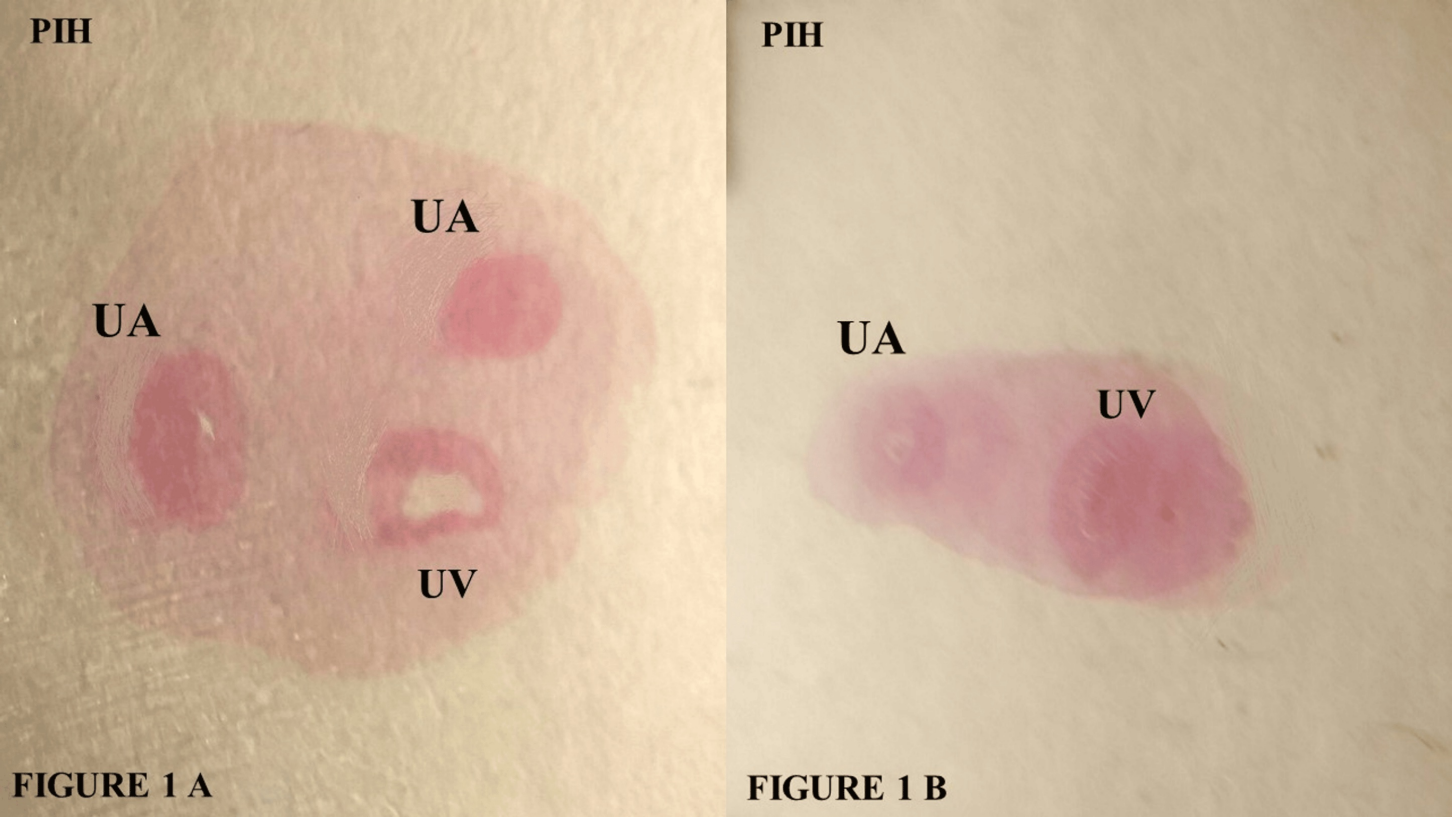
(A) Umbilical artery from a PIH mother, showing two arteries with constricted lumen and a vein with dilated lumen; (B) Umbilical cord from a PIH mother, having a single umbilical artery and a vein embedded in Wharton’s jelly. PIH: Pregnancy-induced hypertension; UA: Umbilical artery; UV: Umbilical vein

Histological examination of the umbilical cord shows several distinct layers under the light microscope. On the surface is a well-defined single layer of simple squamous amniotic epithelium. Deep in the epithelium that comprises the surface of the cord is the mucopolysaccharide substance, Wharton’s jelly. Embedded within the Wharton’s jelly are the umbilical vessels. The vasculature of the umbilical cord is composed of two arteries and a single vein, as shown in Figure 1A. However, sometimes a variable number of umbilical vessels can also be found, with a single umbilical artery being the most common variation, as shown in Figure 1B.

Histology of umbilical arteries

**Figure 2:**
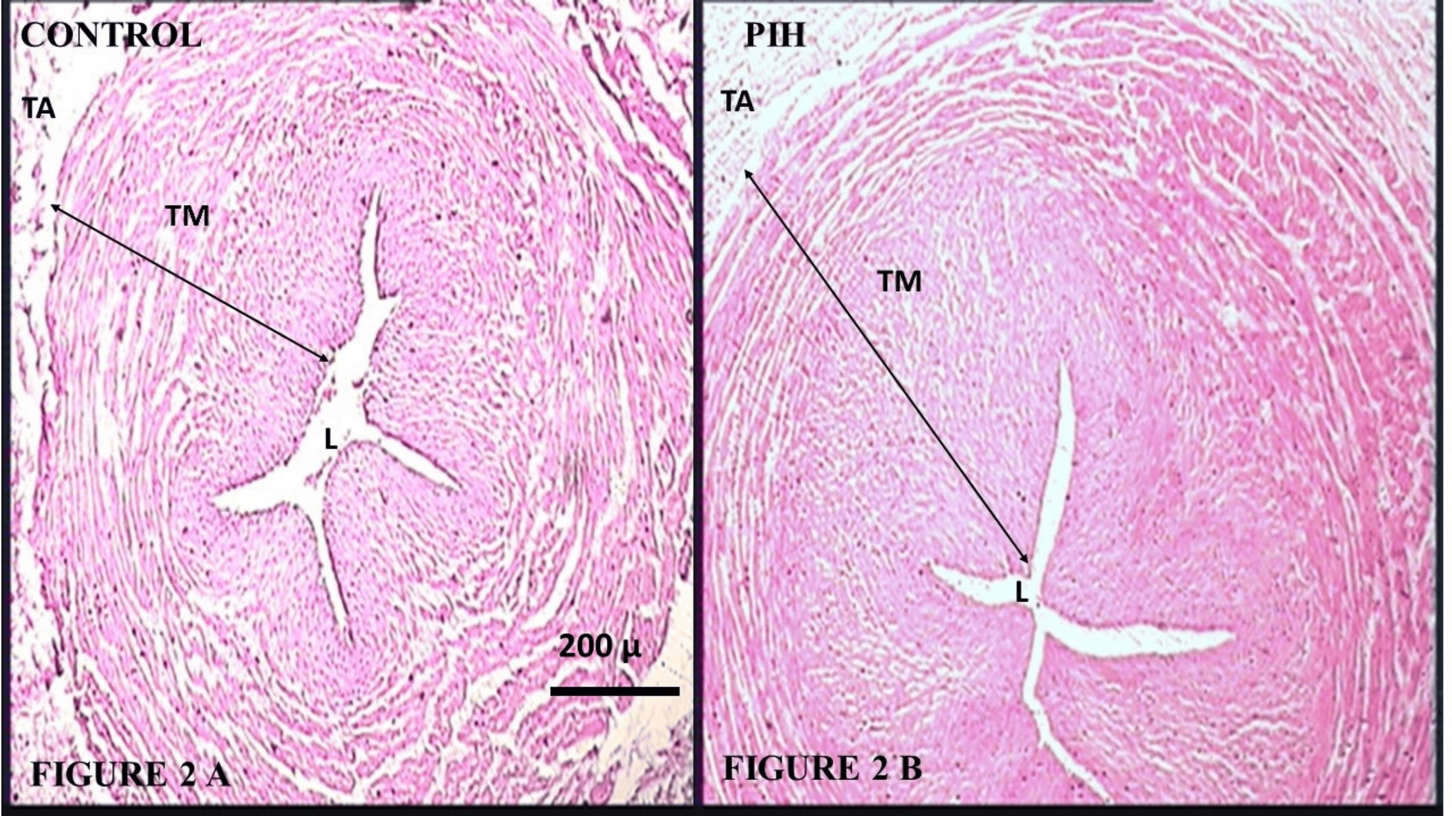
(A) Photomicrograph of umbilical cord from the Control Group showing the lumen and wall of the umbilical artery (H&E, 40X); (B) Photomicrograph of umbilical cord taken from a PIH mother showing narrowing of the lumen and thickening of the wall of the umbilical artery compared to the Control Group (H&E, 40X). TA: Tunica adventitia; TM: Tunica media; L: Lumen; PIH: Pregnancy-induced hypertension; H&E: Hematoxylin and eosin

**Figure 3:**
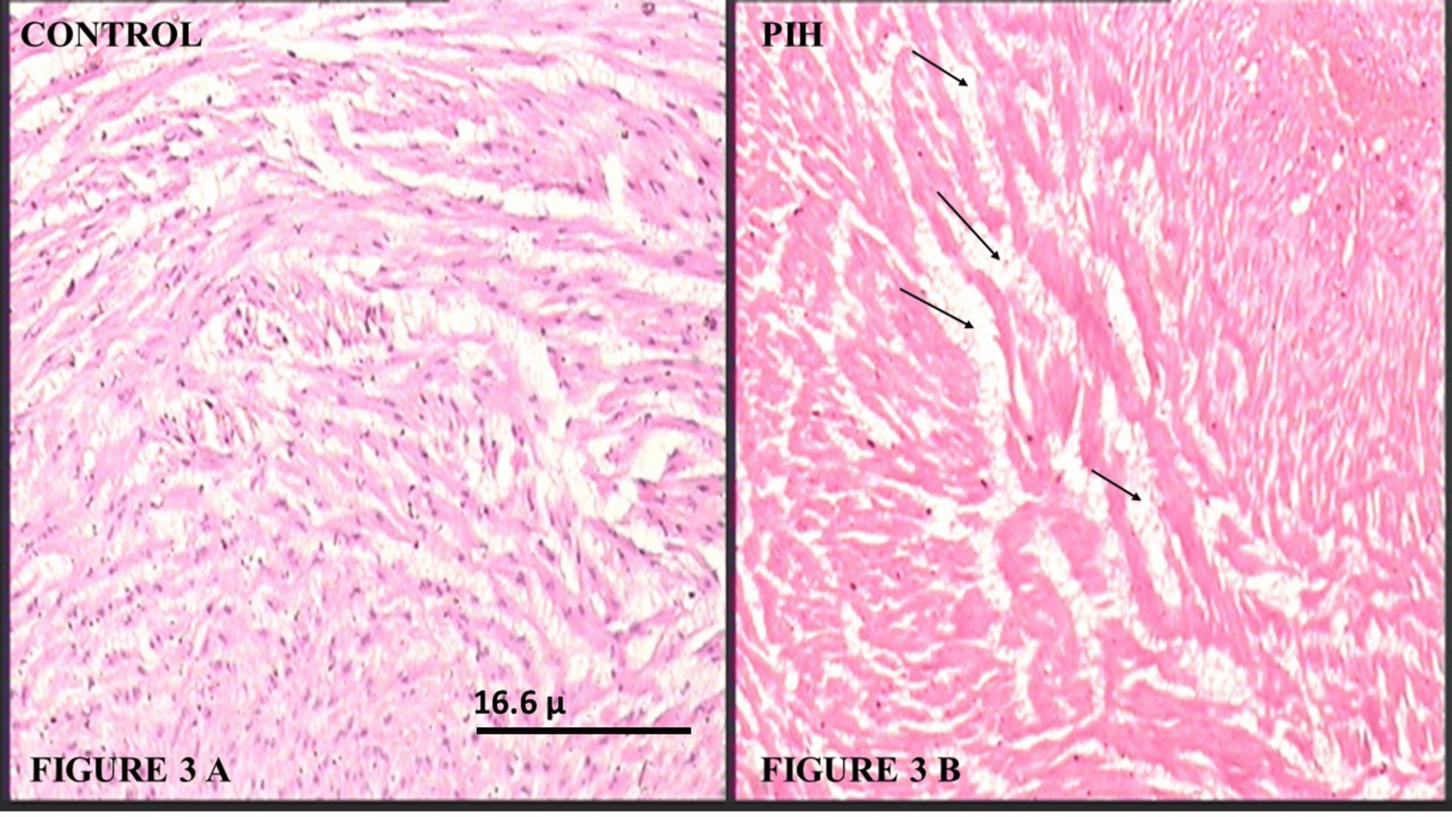
(A) Photomicrograph of umbilical cord from the Control Group showing the tunica media of the umbilical artery (H&E, 100X); (B) Photomicrograph of umbilical cord from a PIH mother showing the tunica media having increased intercellular separation, indicated by arrows, decreased cellularity, and deposition of fibrinoid material in the umbilical artery (H&E, 100X). PIH: Pregnancy-induced hypertension; H&E: Hematoxylin and eosin

Sections of the umbilical cord in the Control Group, shown in Figure 2A, revealed arteries with a star-shaped lumen lined by endothelial cells having prominent nuclei and a well-developed intimal layer, with variable thickenings. The smooth muscle cells (SMCs) were thin, elongated, or wavy, cut in transverse sections. The tunica media was thick, showing an inner layer of longitudinal SMCs, poorly differentiated, and an outer coat consisting of a system of crossing spiraled SMCs. The arterial wall width is depicted in Figure 2A. The tunica media was irregularly arranged, with widened intermuscular spaces, as shown in Figure 3A. Hydropic degeneration was observed in the endothelial cells as well as in the SMCs of the tunica intima and media. No external elastic laminae or distinct tunica adventitia were found.

However, the sections from the PIH Group showed arteries with constricted lumens, and the endothelium was disrupted in many places. The wall of the artery, consisting of the tunica intima and media, showed widening or hypertrophy, which is illustrated in Figure 2B. The wall of the arteries also showed decreased cellularity, deposition of fibrinoid material, and increased intercellular and interlaminar spaces. The normal architecture of the cells and their nuclei was disturbed, as shown in Figure 3B. The hydropic degeneration of endothelial cells and cells in the tunica intima and media was more prominent and numerous.

Histology of umbilical vein

**Figure 4:**
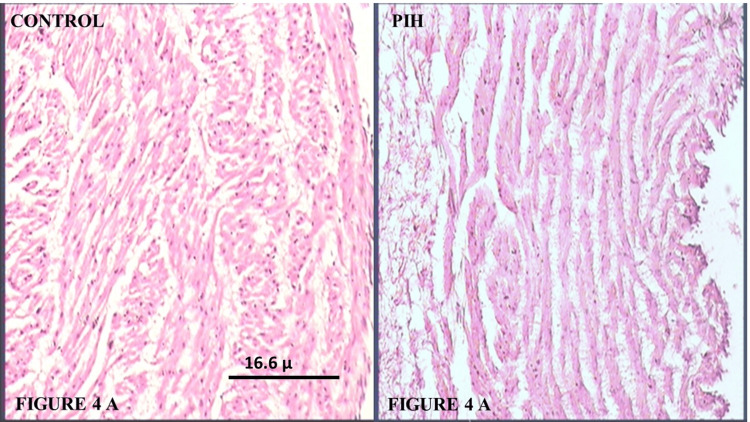
(A) Pictomicrograph of umbilical cord from the Control Group showing laminar arrangement of spindle-shaped smooth muscle cells in the tunica media of the wall of the umbilical vein (H&E, 100X); (B) Pictomicrograph of umbilical cord from PIH case showing disturbed laminar arrangement as well as abnormal architecture of smooth muscle cells in the tunica media of the wall of the umbilical vein. Also, the intercellular gap is significantly increased in comparison to the Control Group (H&E, 100X). PIH: Pregnancy-induced hypertension; H&E: Hematoxylin and eosin

Sections of the umbilical cord from the Control Group, as shown in Figure 4A, show veins with collapsed lumens. The endothelium exhibited a uniform height, with prominently visible nuclei. The wall, consisting of the tunica intima and media, was thinner than in arteries. The tunica intima was much thinner than in arteries. In the tunica media, the SMCs are arranged in circumferential branched laminae, consisting of two or three cells separated by blebs, as shown in Figure 4A. Few groups of longitudinal or oblique SMCs could also be observed.

In the case of sections from the PIH Group, the lumen was found to be dilated. The wall appeared to be thinner than in the Control Group. Again, the intercellular and interlaminar space seemed to have widened, with a marked decrease in cellularity. The blebs separating the muscular laminae appeared more prominent. The hydropic changes in the endothelial cells and SMCs of the tunica intima and media were far more numerous than in the arteries, and also in comparison to the veins of the Control Group. The normal architecture of the SMCs and their nuclei was disturbed. The cells appeared contracted, and the normal spindle shape of the SMCs was lost, as shown in Figure 4B.

Wharton’s jelly

**Figure 5:**
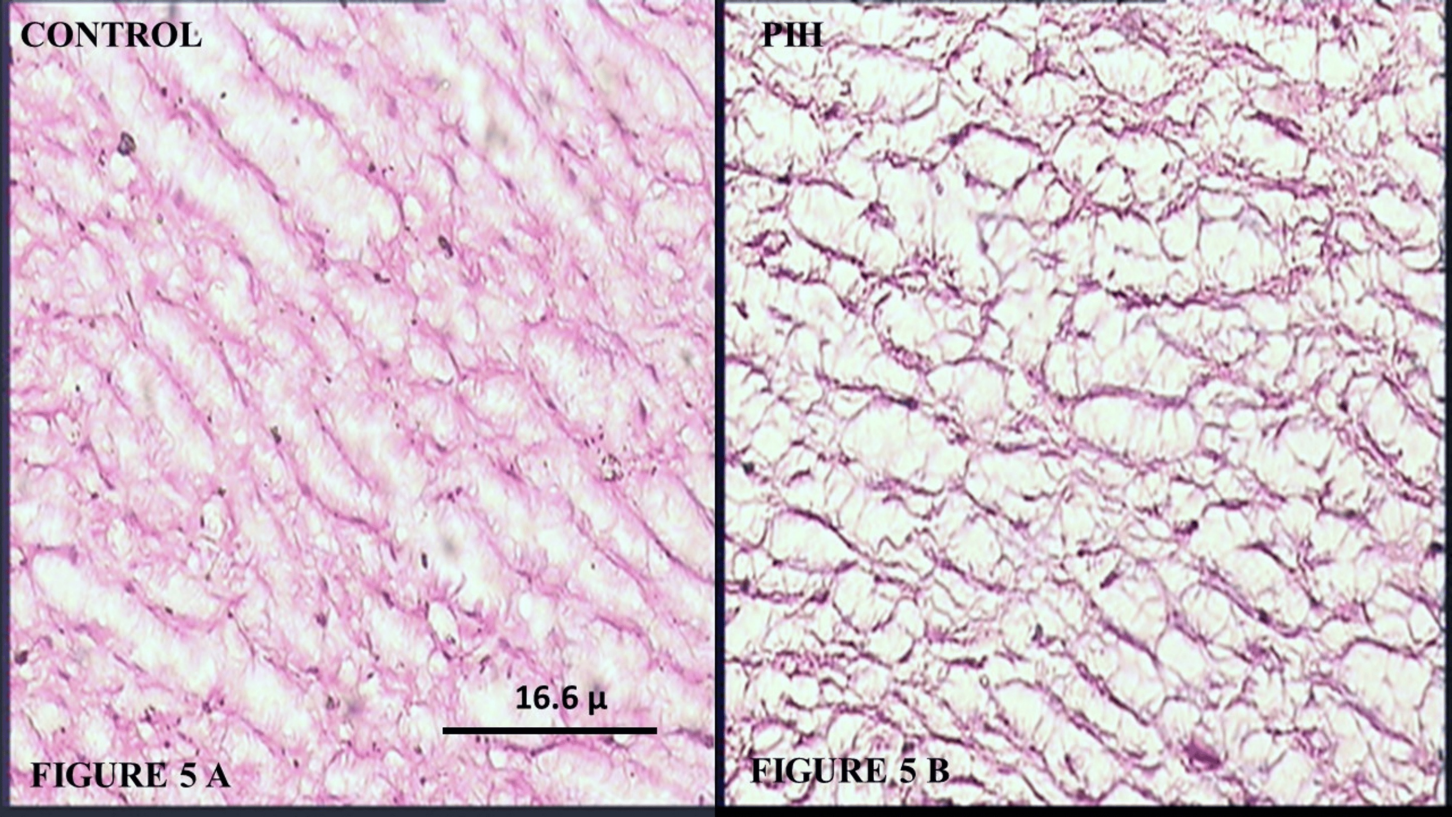
(A) Pictomicrograph of umbilical cord from the Control Group showing Wharton’s jelly (H&E, 100X); (B) Pictomicrograph of umbilical cord from a PIH case showing Wharton’s jelly with decreased cellularity and increased empty spaces (edema) in comparison to the Control Group (H&E, 100X). PIH: Pregnancy-induced hypertension; H&E: Hematoxylin and eosin

In the Control Group, Wharton’s jelly consisted of a mucoid structure, composed of a ground substance of mucopolysaccharides, accumulated around cleft-like territories (“stromal clefts”), occupied by a homogeneous extracellular matrix, devoid of collagen and basal lamina, as shown in Figure 5A. It contained evenly distributed, spindle-shaped fibroblasts with long extensions and varying degrees of differentiation from mesenchymal cells to myofibroblasts. In the umbilical cords from the PIH Group, marked edema of Wharton’s jelly and increased empty spaces were observed, with cellularity significantly reduced, as shown in Figure 5B.

Histomorphometry

The distribution of the thickness of the wall of the umbilical artery and umbilical vein is depicted in Tables 2-3, respectively.

**Table 2:** Distribution of thickness of wall of umbilical artery in μm. PIH: Pregnancy-induced hypertension

S. No.	Thickness in μm	Control cases	Control %	PIH cases	PIH %
1	500-600	2	6.45	0	0
2	600-700	15	48.39	6	19.35
3	700-800	9	29.03	10	32.26
4	800-900	4	12.90	10	32.26
5	900-1000	1	3.23	5	16.13

**Table 3:** Distribution of wall thickness of umbilical vein in μm. PIH: Pregnancy-induced hypertension

S. No.	Wall thickness in μm	Control cases	Control %	PIH cases	PIH %
1	300-400	0	0	8	25.81
2	400-500	0	0	10	32.26
3	500-600	4	12.90	12	38.71
4	600-700	21	67.74	1	3.23
5	700-800	6	19.35	0	0

The mean wall thickness of the umbilical artery and vein in the Control Group and PIH Group is tabulated in Table 4.

**Table 4:** Comparison of mean wall thickness of umbilical artery and vein of the Control Group and PIH Group. PIH: Pregnancy-induced hypertension

Wall thickness (in μm)	Control	PIH	p-value
Vein	667.506 ± 51.31	478.8 ± 76.65	Less than 0.0001
Artery	718.206 ± 75.72	798.715 ± 106.34	0.0002

## Discussion

Preeclampsia is a condition marked by damage to the blood vessels, resulting in a loss of vascular self-regulation, severe vascular spasms, and leakage [2]. Many researchers have studied this condition, offering various explanations for its underlying mechanisms. Biagini et al. suggested that there may be a genetic predisposition to developing preeclampsia [1]. Other potential causes include placental vasoconstriction, intravascular coagulation, and a reduction in maternal blood volume, leading to organ hypoperfusion. Cytotoxic substances released by the placenta may cause damage to endothelial cells. Additionally, an abnormal balance of vasoactive factors in preeclampsia - such as angiotensin, aldosterone, and prostacyclin activity - or disruptions in volume homeostasis can increase vascular permeability and lead to an imbalance between hydrostatic and oncotic pressures [1].

A single umbilical artery is a relatively uncommon occurrence. In our study, the number of umbilical arteries was also affected in patients with PIH, but the difference was not statistically significant compared to the Normal Group. It remains unclear whether PIH contributes to the development of a single umbilical artery, or if the single umbilical artery plays a role in the pathogenesis of PIH. It has been suggested that a single umbilical artery is linked to fetal malformations, IUGR, and higher perinatal mortality in otherwise normally developed infants [10,11]. Previous studies by Inan et al., Pushpa et al., and Yasoob et al. reported a significant reduction in umbilical cord diameter in PIH cases [12-14]. Additionally, Shrivastava et al. observed a decrease in the cross-sectional area of Wharton’s jelly [15].

These researchers observed a significant reduction in both the diameter and volume of the umbilical cord in hypertensive pregnancies, attributing this finding to changes in Wharton’s jelly [14,15]. These conditions often result in restricted fetal growth, making IUGR common in this group. Bruch et al. found that growth-restricted fetuses, whether or not they had abnormal umbilical artery Doppler readings, had smaller umbilical cord cross-sectional areas at delivery compared to normal fetuses [7]. Wharton’s jelly is a metabolically active tissue that plays a role in fluid exchange between the amniotic fluid and umbilical vessels. Lean umbilical cords are frequently associated with torsion, fibrosis of Wharton’s jelly, and thickening of the vascular walls, which can impair fetoplacental circulation, leading to anoxia and fetal death. The morphometric findings in our study were consistent with previous research in both the Control and PIH Groups. Our study showed that the mean thickness of the umbilical artery wall was 718.206 ± 75.720 μm in the Control Group, compared to 798.715 ± 106.336 μm in the PIH Group. This significant increase in arterial wall thickness aligns with the results of earlier studies by Inan et al., Maysoon, Baranwal et al., and Gaikwad et al. [12,16-18].

Histological analysis revealed that both the umbilical arteries and veins were lined with endothelium, with no external elastic laminae or distinct adventitia present. In the PIH Group, the arteries exhibited a narrowed lumen and thickened or hypertrophied walls compared to the Normal Group, consistent with findings from other studies. However, the veins in the PIH Group showed a dilated lumen and thinner walls, which contradicted the results of Junek et al. and Maysoon, who observed no significant histological changes in the umbilical vein in PIH cases [9,16]. Additionally, the endothelium was disrupted in several areas. The arterial walls, composed of tunica intima and media, showed signs of hypertrophy, while the walls of the umbilical vessels displayed reduced cellularity, fibrinoid material deposition, and increased intercellular and interlaminar spaces.

A separation was observed between the muscle cells, caused by an increase in fluid accumulation, which was associated with edema. This edema, occurring in the connective tissue between muscle layers, made it easier to distinguish the layers. Similar to the findings of Inan et al. [12], our histopathological results indicate a narrowing of the vessel lumen and contracted SMCs, which appeared smaller than normal, suggesting a primarily hypoplastic process. Both the SMCs of the arteries and veins exhibited hydropic changes, as did the endothelial cells. This hydropic degeneration, consistent with the destructive effects of hemodynamic stress, was more prominent in PIH cases, as Stehbens et al. also proposed [4].

The deposition of fibrinoid material in the blood vessel walls is considered a nearly pathognomonic feature of preeclampsia [19,20], believed to result from elevated blood pressure [20]. In our study, we observed fibrinoid material deposition in the walls of the umbilical artery in the PIH Group, compared to the Control Group. Proposed explanations for this deposition include the precipitation and thickening of fibrin or other blood derivatives, collagen necrosis, coagulation of ground substance, or a combination of these processes [21]. Our findings are consistent with those reported by other researchers. In hypertensive pregnancies, edema of Wharton's jelly is evident due to fluid accumulation within its matrix. Inter-endothelial junctional protein complexes facilitate the metabolic diffusion of fluids and proteins from the blood vessel lumen to the vessel wall and Wharton's jelly. Increased permeability of endothelial cells in hypertensive conditions disrupts these junctional protein complexes, leading to Wharton's jelly edema. Shanklin and Sibai proposed a similar theory [22]. It can be concluded that the reduction in Wharton's jelly area is due to either hypoplasia or decreased hydration. The accumulation of sulfated glycosaminoglycans (GAGs) in the extracellular matrix of Wharton's jelly is reported to influence the biology of umbilical cord tissue. High concentrations of GAGs and proteoglycans surrounding collagen fibers can affect the solubility of the collagen, making it less soluble and the jelly more compact, which, in turn, impacts the umbilical cord’s mechanical properties and its macroscopic appearance [2].

## Conclusions

The thickness of the umbilical artery wall in the PIH Group was significantly higher than in the Control Group, while the umbilical vein wall thickness was notably reduced. Histological examination revealed significant changes in the umbilical cord of PIH patients, including contracted lumens and thickened walls in the umbilical artery, along with fibrinoid deposits and hydropic changes. The umbilical vein displayed a dilated lumen, disturbed smooth muscle architecture, increased intercellular space, and thinning of its wall. Wharton’s jelly showed signs of increased edema and decreased cellularity. These changes suggest that preeclampsia-induced maternal hemodynamic alterations contribute to fetal hypoxia, hypertension, and increased vascular resistance, leading to morphometric changes in umbilical vessel walls that may indicate IUGR.

This study addresses a gap in the literature by providing histomorphometric evidence of umbilical cord alterations in PIH patients. The observed changes in umbilical vessel walls may serve as potential markers for IUGR in term preeclamptic pregnancies, which could improve antenatal care and risk assessment. The study also highlights the pathophysiological link between preeclampsia and fetal vascular alterations. However, limitations, such as a small sample size, single-center design, and exclusion of preterm pregnancies, must be considered. Future research with larger, multicenter studies is needed to validate these findings and explore the clinical application of these structural changes as early markers for fetal complications. Incorporating stereological principles in future studies could ensure more accurate and unbiased measurements.
